# Redressing or entrenching social and health inequities through policy implementation? Examining personalised budgets through the Australian National Disability Insurance Scheme

**DOI:** 10.1186/s12939-017-0682-z

**Published:** 2017-11-06

**Authors:** Gemma Carey, Eleanor Malbon, Daniel Reeders, Anne Kavanagh, Gwynnyth Llewellyn

**Affiliations:** 1 Centre for Public Service Research, UNSW Canberra, Canberra, Australia; 2 School of Population Health, University of Melbourne, Melbourne, Australia; 3 Centre for Disability Research and Policy, University of Sydney, Sydney, Australia

## Abstract

**Background:**

Increasing attention is being given to political agenda setting for the social determinants of health. While designing policies that can improve the social determinants of health is critical, so too is ensuring these policies are appropriately administered and implemented. Many policies have the potential to entrench or even expand inequities during implementation. At present little attention has been given to this in the social determinants of health literature.

There is an international trend in the personalisation of funding for care services, from the National Health Service in the England to the Brukerstyrt Personlig Assistanse in Norway. Part of this trend is the Australian National Disability Insurance Scheme (NDIS). The NDIS has the potential to secure gains in health for hundreds of thousands of Australians living with a disability. However, policies are only as good as their implementation.

**Methods:**

As part of a longitudinal study on the implementation of the Australian NDIS, we conducted a systematic document search of policy documents pertaining to the Scheme on the websites of government departments with auspice over the design and implementation of the scheme with the aim of examining issues of equity.

**Results and discussion:**

Scheme architects have argued that the NDIS has the potential to replace a piecemeal and fragmented set of state-determined services with an empowering model of user choice and control. However, without careful attention to both existing inequities and, diversity and difference across populations (e.g. different disability types and different localities), market based approaches such as the NDIS have the serious potential to entrench or even widen inequities.

**Conclusions:**

The research concluded that ‘personalisation’ approaches can widen inequities and inequalities unless careful consideration is given at both policy design and implementation stages.

## Introduction

It is now well established that many of the key drivers of health reside in our everyday living conditions [1, 2]. In the last four decades a large volume of research evidence has been developed which documents the varied ways in which social, economic, political and cultural environments impact upon health [2, 3]. Many of the societal level factors that affect health – known as the ‘social determinants of health’ – are social issues which exist outside the health sector such as housing, education and employment. Of particular concern, are inequities or disparities in health and wellbeing that occur because of the unequal access to social and economic resources, and differences in exposure to certain conditions [3–6]. Hence, unequal social conditions have been found to lead to differential and unequal health outcomes [3]. Moreover, some have argued that the existence of health inequities and other forms of social and economic inequity affect the whole population’s health not just that of the most disadvantaged groups [7].

Increasingly, research attention has been given to political agenda setting for the social determinants of health (i.e. how to identify the right policies for addressing health inequities, and how to ensure they receive political support) [8–16]. While designing policies that can improve the social determinants of health is critical, so too is ensuring these policies are appropriately administered and implemented [17, 18]. Even universal policies (i.e. those which cover the the whole population [19] and are argued to protect against inequities [3, 20, 21]) have the potential to entrench or expand inequities during implementation processes without careful attention to existing inequities, and how they might interact with the policy being implemented [19].

This paper is concerned with the implementation of a national disability policy in the Australian context. Globally, one billion people (15% of the world’s population) are estimated to live with a disability [22]. More than four million Australians report having a disability, including over 2.2 million adults of working age (16% of the working age population) and 4.3 million in total (18.3% of the whole population) [23]. These individuals have poorer health and social outcomes than non-disabled people [24], making disability an important social determinant of health. Research has shown that Australians with disabilities are also likely to be exposed to well-established adverse social determinants including social and economic exclusion, unemployment, unaffordable housing, and weak social networks [24–28]. Hence, evidence indicates that the poorer health of people with disabilities is not simply a product of their impairment but may, in large part, be due to the circumstances in which they live [24, 27]. These circumstances have been shown to not just include the immediate living conditions of individuals, but also the broad social and political structures (i.e. welfare state models) in which they reside [29, 30]. Additionally, social and economic outcomes appear to be worse for people with mental health problems, acquired brain injury and people with intellectual disabilities than for people with physical disabilities or sensory and speech problems, indicating the need to understand interactions between living circumstances and particular health conditions and impairments [24, 27]. How policies are designed and implemented – and the ways in which they impacts the lives of people with a disability – therefore has profound consequences for health outcomes and in turn social and health inequalities.

The Australian National Disability Insurance Scheme (NDIS) draws on policy trends in the UK and Europe [31–33] towards ‘personalisation’ of social services. Under these approaches funds are given directly to people with a disability so they can purchase services and supports that best meet their needs, rather than standard ‘one size fits all’ programs [34].

It has been argued that the NDIS has the potential to secure gains in health for hundreds of thousands of Australians living with a disability [34]. The different model of care (i.e. personalisation) used by the NDIS is meant to enable access to more appropriate services, empowerment, social and economic participation – all of which are known social determinants of health [2, 3]. However, policies are only as good as their implementation [17, 35]. As noted by Carey and Friel (2015), from a population health perspective, there has been relatively little study of the complex policy frameworks and administrative layers through which policies that impact the social determinants of health are implemented.

In this paper we provide an overview of the Australian National Disability Insurance Scheme (NDIS). We then analyse the potential of the scheme to redress, entrench or extend social (and in turn health) inequities, using design documents and reports released during implementation. Our analysis aims to: (a) demonstrate how the NDIS and its international counterparts might contribute to or reduce social and health inequalities and (b) develop knowledge of how social policies can inadvertently extend health inequalities unless careful consideration is given at both policy design and implementation stages. We argue that policies like the NDIS demonstrate that the way that funding is allocated, and not simply the amount of funding allocated, matters for health outcomes and inequalities.

## Background

Over the past 30 years there have been major shifts in the way government(s) deliver public services. Increasingly, governments aim to give citizens greater choice and control of the public services they utilize [36]. As a result, we have seen the creation of various forms of public sector markets [37, 38]. Here, governments create ‘markets’ through contracting and tendering processes or by individualized care budgets (i.e. where individuals are given money to purchase services that meet their needs) [36, 39, 40]. This has occurred in the UK, Germany and the Netherlands to name a few [41]. By enabling choice and control, proponents believe that personalized budgets and care markets improve wellbeing [36]; rather than utilizing a ‘one size fits all’ service, citizens can (in principle) choose services that best meet their needs. The NDIS represents a further extension of these principles by governments internationally; the NDIS is the first major social policy in Australia that utilises personalized budgets and is subsequently creating a public service quasi-market for disability care [34].

Individual budgets (provided within personalization approaches to policy) in the disability policy first emerged in the United Kingdom in adult social care, inspired by earlier social movements in the US [40]. This was part of both a fight for redistribution and recognition by disability advocates [42]. The personalisation agenda has also emerged from broader pressures on welfare states. Faced with a range of fiscal and social pressures, we have seen shifts in many industrialised countries away from collective social welfare provision in favour of markets and ‘self-directed care’ [43]. However whether these approaches lead to improvements in people’s lives is still a matter of debate [44, 45].

The Australian National Disability Insurance Scheme (NDIS) is Australia’s first serious foray into personalised budgets [46]. The NDIS was passed in legislation with (rare) bi-partisan support in 2013 with broad public and political support [47]. Under the NDIS, approximately 460,000 individuals who have a significant and permanent disability will receive personalized funding budgets [34, 48]. These funding packages are determined on the basis of need by a newly established agency – the National Disability Insurance Agency in conjunction with scheme actuaries who determine pricing and, to some extend, the size of packages (see [49, 50] for more information on the implementation of the NDIS). Individual packages are on average between $10,000 and $30,000, but can range into the hundreds of thousands of dollars [51]. By providing choice and control, the Scheme is expected to deliver benefits to these individuals, their carers and families – extending the potential benefits of the scheme to hundreds of thousands more individuals [34].

The scheme will be fully implemented across Australia by 2019, including in urban, rural and remote localities and across a diverse range of disability types [34, 48]. Scheme rollout was commenced in seven trial sites which targeted different population groups. Some trial sites prioritized particular types of disabilities, others age, while some were geographical [52]. The scheme shifted to national roll out in 2016 [49], At present 100,000 Australians are signed up to the scheme, with rollout determined by geographical region [53]. Under the new ‘personalised’ model individuals are given funding packages, determined by their level of need and self-defined goals, with which to purchase services [34]. This is anticipated to secure choice and control for the person with a disability.

As the architects of the NDIS, the Australian Productivity Commission perceived the previous disability care system to be a piecemeal and inequitable system which has negatively impacted those living with a disability [34]. Previous to the NDIS, disability care services were funded according to a commissioning model in which community based service providers competed for 1–5 year blocks of funding from which they ran a variety of services [34]. In addition to commissioned community service providers, private service providers functioned in a market based system with care given to those who could afford to pay from private funds. Such private service provision options continue today. Advocacy for a move to the NDIS focussed on changing the balance of power in decision making about the lives of people with disability, ostensibly to create a more equal disability care system [34, 47]. Here, advocates were drawing on debates in social policy which demonstrate that when benefits and supports are generic (i.e. one size fits all) they produce unequal experiences and outcomes for users because they fail to take account of differences in need [19, 54, 55]. Moreover, the previous commissioning arrangement caused a privileging of professional’s (public servants) opinions in decision making about “fundamental elements of disabled people’s lives such as where and how they should live, whether or not they should work, the type of school they should attend, the type of support they need and whether or not they should become parents.” [47, p17] The previous system was widely regarded as insufficient and inequitable because of its highly fractured and ‘one size fits all’ approach [34, 47, 50, 56, 57], which has been shown in public health to be inconsistent with the needs of vulnerable groups [21].

While there are multiple options for structuring a Scheme to allow for greater choice and control in care delivery, for example pay-for-performance or spot contracting, the decision by the Australian Government, informed by the Productivity Commission and a large community based campaign, was for an individualised payment system [34, 47].

The NDIS is positioned as differing from the previous arrangements due to a focus on choice and control: the Productivity Commission claims that previous disability services led to “disempowerment, no choice, a take or leave it attitude of service providers and retribution for leaving, or complaining, about a service that is unacceptable, inadequate or unsatisfactory” [34]. In addition to the focus on choice and control the National Disability Insurance Agency, tasked with implementing the Scheme, has stated that “at the heart of the NDIS is equity… We are finally moving away from a fundamentally flawed and inequitable system. A system… described as ‘under-funded, fragmented, unfair and inefficient’” [56].

Whether the NDIS will prove to be a more equitable system than the previous arrangements or whether it will simply perpetuate a different form of inequality remains unknown. At the outset, it is worth noting that there are important questions regarding the equity of service, support and outcomes between the 460,000 eligible individuals (i.e. those with a permanent or likely to be permanent and severe disability) and that some four million ‘ineligible’ individuals who may receive supports through the Australian National Disability Strategy and other sources, but less than participants [for more information see 57]). While this is not central to this paper, this raises questions about the equity of disability funding and support in Australia more broadly [see 58].

While personalised budgets have been used in other countries, the Australian experience is unprecedented in several important ways. Firstly, the geographical spread outstrips that of other countries. Secondly, in the UK individuals have the choice to opt into personalised schemes, however in Australia the scheme is compulsory for eligible individuals [46]. Hence the scale of the NDIS is broader and deeper than its international counterparts (such as the National Health Service in England which has utilised personalised approaches in aged care and disability, and Brukerstyrt Personlig Assistanse in Norway, and similar programs in Scandavia and Denmark [31, 32, 59]), offering important opportunities for learning.

## Methods

As part of a longitudinal study on the implementation of the NDIS, we conducted a systematic document search of policy documents pertaining to the NDIS on the websites of government departments with auspice over the design and implementation of the scheme. These are: Commonwealth Department of Social Services, Coalition of Australian Governments [COAG] and the National Disability Insurance Agency. We also included the Australian Productivity Commission which, while not a government department, has played a highly significant role in advising the government on the design of the NDIS – their report is considered to be the ‘blueprint’ for the NDIS and its implementation.

This search captured both major policy design and implementation documents (*n* = 25). Documents range from key design documents (i.e. Productivity Commission 2011), to implementation reports [e.g. 60] and reviews [e.g. 52] (see Appendix for full list).^1^ Many (*n* = 21) pertain to operational aspects of the scheme, e.g. financial reports and numbers of participants enrolled. We identified four critical documents that spoke to the question of equity and provide insight into the principles and philosophy of the NDIS. These are; the Productivity Commission’s design and implementation guide for the NDIS [34], the proposal, consultation report and final plan for a National Quality and Safeguards Scheme [61–63] and a key document on the development of the NDIS market [64].

The five documents identified as relevant were then analysed thematically with the goal of locating decisions made during design or implementation that had consequences (positive or negative) for social and health inequalities. We used the empirical evidence base on the social determinants of health inequities as a guide to assist in identifying policy decisions which could impact inequities and inequalities. In particular, we were guided in our analysis by the Commission on the Social Determinants of Health and the Marmot Review of Health Inequalities [2, 3]. In doing so, we sought to critically analyse how the NDIS could modify, redress, entrench or extend social (and in turn health) inequities.

## Findings

Three interrelated areas pertaining to differences in care and outcomes emerged from our analysis particularly regarding differences in ability to exercise choice. These related to disability type geography, geography and issues related to disability markets. While not exhaustive, these represent the areas of most significant vulnerability for entrenching or creating inequalities and inequities. All three intersect with the question of whether markets can deliver high quality and equitable services to citizens.

## Differences in choice and control of services

As noted, the NDIS is part of a shift in the way governments fund services to their citizens, which has been building for 30 years [37, 38, 65]. The push behind this trend has been to give citizens more choice and control of the services that they can access. This is in part a reaction to the perception that ‘universal’ funding approaches do not sufficiently acknowledge or address diversity [65]. Traditionally, governments have provided services directly to citizens – offering a universal or ‘one size fits all’ approach, as demonstrated by the previous disability care arrangements in Australia. However, although universal supports are an important precursor for equity they cannot, in and of themselves, achieve it because of their inability to account for differences in need [41, 54]. Determining how to account for diversity has become a major challenge for policy [20, 21, 41, 54]:

“diversity conflicts with both universal inclusion and universal allocation. This goes beyond gender and ethnicity. Each individual is increasingly seen as differing from other fellow citizens. It has been argued that universalism has become problematic and lost its appeal to many citizens. The growth of middle-class influence and increased cultural diversity… has strengthened social policy discourses based on ideals such as diversity, participant and freedom of choice” [41].

In response, governments have shifted towards what is known as block funding – where organisations outside of government are given resources to deliver a particular program. This approach is argued to provide citizens with a greater range of choices [36], however the effectiveness of this is contested [66]. The next phase of reforms has gone further, focused on ‘personalisation’, otherwise known as ‘individualisation’ of funding. Proponents argue that this is the best way to deal with the diversity of needs which exist within the population – allowing individuals to tailor their supports to their circumstances [67].

Under the NDIS, moving to a system of personalised planning and allocation of funding packages for disability supports, it is argued, will radically change the structure of care provision, emphasising individual choice for purchasing supports in a way that ‘block contracting’ arrangements have traditionally struggled to achieve [34, 52, 60]. In the UK, these approaches have been said to have “the potential to improve the social status of disabled people by transforming their identity from that of passive service recipient to active employer” or (as is more common in Australia with people choosing not to self-manage their funds) an active purchaser through the setting of personal goals [42]. From a government perspective, this change in relationships in the context of a disability service market is seen as a way to deliver more efficient, responsible and innovative services than is achievable with large public bureaucracies [65, 68]. However, as Needham [69] suggests “personalisation is an agenda in which policy roll-out is racing ahead of the evidence base—spreading into new services before earlier pilots are concluded”. From a social determinants of health perspective, more thought needs to be given to the ways in which personalisation under schemes like the NDIS could entrench or extend social and health inequities. This means examining existing inequities that could be compounded by introduction of the NDIS both within and between groups (i.e. on the basis of disability type, gender, culture, age or locality). Some of these challenges were clear at the design stage of the NDIS and noted in early documents as an issue that needed to be accounted for and redressed in implementation. For example, geographical locality was raised by the Productivity Commission, in the first design documents for the scheme, as a barrier to choice and control for some individuals [see, for example, 34]. Other potential inequities have emerged as implementation progresses, such as age and disability type.

### Differences between disability type

While the principles of empowerment, choice and control are important to health [3], we need to recognise differences in people’s abilities to exercise this choice and control. While the evidence regarding personalisation is in its infancy [40], currently it indicates that in some contexts personalisation can lead to greater satisfaction and continuity of care and a more effective use of public resources [70, 71] – thereby redressing inequities between people with and without a disability. However, in the UK take up of opt-in personalisation schemes for managing individual budgets and care has been relatively low [40]. Williams and Dickinson (2015) argue that this cannot be put down to a lack of interest, but rather reflects the capacity of individuals to engage in personalised care and of professionals and social networks or carers to support people to engage. Williams and Dickinson (2015, p.5) note that “personalisation policies and approaches have not been welcomed by all social workers who play an important role in the brokerage of these types of arrangements”. Critically, Williams and Dickinson (2015, p.5) have found differential take up and outcomes between amongst those accessing such supports.

To date, evidence from the UK has shown that individuals with physical disabilities are able to take better advantage of these opportunities than those with intellectual impairments. In the latter, good outcomes appear to depend upon strong advocacy or brokerage support – highlighting the critical need to gain support from professionals working within the scheme [33, 40, 42]. In the UK, Riddell et al. [42] found that the top users of individualized funding and management are people with physical and sensory impairments, with people with mental health problems and neurological impairments the least likely to opt in. This is consistent with the literature on health service usage, whereby those who are more disadvantaged are less likely to access services or support and receive less benefit when they do [72, 73]. Similar findings exist with regard to the Australian accident and injury compensation schemes (upon which the NDIS is based); higher take up is found amongst those with physical disabilities rather than neurological impairments such as acquired brain injury [74]. This suggests that personalization and individual budgets can widen inequities between people with different types of disabilities.

A critical difference between the UK and Australian context is that in Australia personalization for those deemed eligible is not a choice – all eligible individuals will be participants in the NDIS. Around 70% of eligible participants have an intellectual disability or autism and related disorder [60]. This enforced participation has the potential to be favourable in terms of equity, as the extra effort required to opt into a scheme is lessened. Yet participation of individuals with different types of disability does not guarantee equitable outcomes; the benefits of personalised funding are also dependent upon other social determinants, such as the presence of social support, education level and income [39].

The extent to which the benefits of personalized funding are realized depends upon the advocacy and support networks individuals have access to [33, 40]. Under the NDIS, individuals must have the ability to negotiate and define goals and plans, and where this is not possible an advocate negotiates on their behalf. For some individuals, Williamson and Dickinson (2015) have found that they neither want nor expect to have to direct their own care. This makes intuitive sense, as being able to maximize the gains of personalized care budgets requires skills in managing them as well as navigating new systems. Willingness and ability to self-manage and control care is likely to be a major challenge for young people as a result of the administrative burden with disability who make up a large proportion of participants [75].

The UK evidence demonstrates that individuals with significant supports in place prior to personalization (i.e. financial and interpersonal) are more likely to experience benefits than those who do not [33]. This suggests that those who are already marginalized or of low-socio-economic status may benefit least from the NDIS (though they still may benefit more than under the previous system because of the overall investment in disability awareness, though this is yet to be seen or tested). This is consistent with the inverse care and prevention law [76], a problem which has plagued population health interventions [1, 77]. Here, individuals who need to gain the most from health interventions actually gain the least. The inverse care and prevention law has been evident across diverse areas of health promotion activity [78, 79], the most famous case being smoking cessation campaigns which have been found to have greater take up amongst high socio-economic groups [72]. Link and Phelan note, ‘resources’ (whether financial or social) are fundamental causes of health and thereby link to multiple disease outcomes through different pathways. These same resources determine the ability of individuals to exercise choice, control and navigate service systems – also linking them to poor service use and/or satisfaction. The inverse care and prevention law can be seen in Needham’s [33] work in disability, which has shown that there is little supporting evidence that personalization efforts have a positive effect on social inclusion or income. She argues that “evidence highlights the dangers of inequity between those with financial and social resources to supplement their use of budgets and those without” [33, 42]. That is, those who have more resources are more likely to reap the benefits of personalization than those without (consistent to the inverse care and prevention law). Considering the significance of the NDIS as a major health and welfare reform, careful attention needs to be given to whether the NDIS plays out according to the inverse care and prevention law and exacerbates inequities and, if so, how to mitigate this.

Currently, the implications of personalized funding and individualized budgets for equity under the NDIS are unclear, based on the differential ability of individuals to engage in exercising the choice and control supposedly afforded to them by such an approach [33, 40, 80]. This may be on the basis of education, lack of supported learning, or a lack of a market from which to choose (which we will discuss below). It is worth noting that at this early stage of implementation within the NDIS, participants are currently choosing from a defined (and costed) set of services, akin to a ‘menu’ of services, which has implications for the flexibility of funds being provided and the goals that can be set by individuals [75, 78].

Personalised budgets have the ability to advance health through empowerment and better utilization of care and support resources. However, they also have the potential to entrench or expand existing inequities, and early evidence indicates that this is a real risk for the NDIS [79, 80]. For example individuals with intellectual disabilities – who are already more marginalized – appear to fare worse under such arrangements unless they have advocates or strong support networks (i.e. exercising less choice and control and experiencing worse health outcomes) [33, 42]. Moreover, socially isolated people or those without strong support networks and resources to supplement personal budgets also do not reap the same benefits as those who do have strong supports. In this instance, there is a risk of significantly extending inequities between these individuals and other groups accessing personalized care budgets (and the rest of the population) – consistent with the inverse care and prevention law.

### Differences emerging from disability service and support markets

The NDIS is underpinned by a market based approach [34, 64]. This market includes services and supports – some of which may be disability specific while others may be more general or mainstream. Internationally, markets have been treated by governments as a panacea for social policy challenges: “choice and competition as a model for public service delivery… fulfils the principle of autonomy, and promotes responsiveness to users’ needs and wants; it provides incentives for providers to provide both high quality and greater efficiency; and it is likely to more equitable than the alternatives” [36]. Hence, for both sides of politics, ‘choice and control’ of public sector services through markets are seen as a way to gain economic efficiency, while enabling citizens to have a more empowered relationship with the State. However, governments find markets of all types notoriously difficult to regulate and manage in predictable and reliable ways and markets, by very nature, tend to produce winners and losers in terms of both operators and users [19]. That is, citizens need to have the right capabilities to exercise choice and control [58, 81]. This is noted as an area of concern in the two documents on market safeguards, yet it remains unclear whether (and if) differences in capabilities can be overcome: “central to the framework are developmental safeguards designed to make sure participants have the capabilities and supports to be able to choose quality supports and to build good and safe lives” [82]. Yet, as Soldatic [58] notes, this will be challenging for people who are marginalized and experience multiple and complex forms of disadvantage.

While the NDIS is anticipated to have a highly diverse and well-functioning market by full scheme implementation in 2019, how exactly this market will function and what role government will have remains undecided [61, 82]. This is again reflective of the international literature; questions about how to develop, oversee and ensure the effectiveness of public sector markets remain vexed [45, 68]. As noted earlier, the scope and scale of the NDIS market makes its development particularly challenging. The market must cover all types of disability as well as account for enormous geographical spread, in addition to other types of diversity (e.g. culturally and linguistically diverse communities and people with low literacy). This presents two critical challenges to ensuring equity: ‘thin markets’ and market failure. Thin markets emerge when there are not enough providers in a public or private market for it to function as intended [83]. Thin markets have both a low number of buyers and a low number of sellers, and may also suffer from price volatility – a combination of characteristics that leads to market inefficiencies or failure (i.e. complete market collapse where no providers are left or significant gaps. Thin markets and market failure are more likely to occur in regional areas or for those with highly specialized needs, as noted in implementation documents: “The risk of market failure remains an issue in many areas. Market failure can include the failure of individual suppliers or organisations, localised market failure or more systemic failures related to scenarios such as predatory practices, unbalanced supply and demand, unbalanced information about support, consolidation, decrease in participant choice, and decrease in the quality of service choices.” [64].

In the UK context Gash [45] .has shown that to guard against inequities emerging from public sector markets governments must participate in: engaging closely with users, provider organisations and others to understand needs, objectives and enablers of successful delivery; setting the ‘rules of the game’ and allowing providers and users to respond to the incentives this creates and constantly monitoring the ways in which the market is developing and how providers are responding to these rules, and the actions of other providers. Governments must also be involved in adjusting the rules of the game in an attempt to steer the system (much of which is, by design, beyond their immediate control) to achieve their [government’s] high-level aims [45].

The quality and safeguard reports indicate that the role the Australian government will play in terms of ‘market stewardship’ is yet to be determined [82]. The nuances of that decision will have far reaching consequences for health equity.

### Widening inequities between groups on the basis of locality?

Rather than one national market, the NDIS actually requires many local markets that account for geographic diversity. The NDIS acknowledges that developing these markets will take time: “developing a strong, contestable market for disability supports is a long term project” [84]. In inner urban centres this may be achievable, but potentially less so in outer urban areas particularly with regard to Indigenous, culturally and linguistically diverse communities with potentially low prevalence of particular types of disability. In rural and, particularly, remote areas ‘thin’ markets (i.e. where only one or two providers exist) are likely to emerge:

“Where there are thin market segments, such as rural and remote areas, providing choice will be more difficult and may require a greater level of market facilitation. It should also be acknowledged that there may be high personal and economic transaction costs to change providers, and these should be minimised.” [64].

In urban or peri-urban areas low prevalence of disability (or specific types of disability or particularly challenging situations with few or no support providers) may also present challenges in terms of thin markets. Thin markets are also susceptible to market failure, where no new providers enter the market place due to high costs of entry or lack of business prospects, and existing providers are challenged by being paid retrospectively for business, gaining the necessary breadth and depth of expertise and business costs running higher than the funds collected via individuals. This is particularly risky while prices are set by the government (something which is hoped by policymakers, in time, will change) [34, 64]: “Ultimately, the pricing role of the Agency would diminish as the market developed, and this could allow disability services to even more closely resemble the economy-wide service sector” [34].

In the case of market failure or thin markets, individuals already disadvantaged geographically are unlikely to be able to exercise true choice and control through personalisation. It was mooted in the initial report recommending the NDIS that some ‘block funding’ by governments (i.e. the traditional contracting and procurement processes that currently exist) may continue: “block funding may continue in certain circumstances, such as in building community capacity, pilots of innovative services, in some rural areas where markets might not support the provision of any service, and where there is a need to build longer term capacity, such as Indigenous-specific services” [34]. Building capacity falls within the ‘Information Linkages and Capacity Building’ (ILC) component of the NDIS, which recognises that not all needs can be met through personalised funding and that some degree of ‘whole of community’ capacity building is required. The ILC component may therefore act to prevent inequities between areas and or groups through supporting communities and mainstream services to become more inclusive [85]. However, concerns have already been raised about the capacity for ILC supports to be delivered given the potential workload associated with those carrying out this role (known as ‘Local Area Coordinators’), who support NDIS participants with planning and identifying mainstream and disability specific services [86].

Additional or continued block funding has been suggested as a potential (last resort) means by which to address market inequities [34]. Markets, in the view of the Commission, produce better outcomes than hierarchical public service systems:

“The scope for full competition may not always be present when suppliers have market power, consumer knowledge is poor, where services are complex, or where the market context would be likely to lead to distorted consumer decisions. Markets may also take some time to develop, as will the capacities for making informed choices by people with a disability and their families (hence the need for supporting people in implementing self-directed funding). However, choice among specialist disability services may often still produce better outcomes even where markets are imperfect” [34]

Hence, the proposed market mechanisms may exacerbate inequities between urban, rural and remote areas. Under these conditions, the government suggests that block funding should continue:

“In such cases, the National Disability Insurance Agency should block fund suitable service providers to work with local communities to deliver disability supports to Indigenous Australians. This approach will be particularly necessary in remote areas. In doing so, it should work with existing government agencies, Indigenous advocacy groups and other funded service providers” [34].

However, if the core of the NDIS is to offer empowerment through choice and competition, there is a need to recognize that not all individuals will have access to robust or functioning markets by which to exercise this control. Moreover, block funding could limit innovation with regard to services. In essence, two schemes may emerge – one in urban areas with robust markets, and a second (lesser) scheme subsidized by government in rural and remote areas that continues to offer little choice. From a social determinants of health perspective, individuals likely to access these continued block-funded services are also more likely to already be experiencing other forms of inequity and/or disadvantage. For example, individuals living in Australian rural and remote areas have lower incomes, and worse health and wellbeing [87]. They also experience more challenges accessing health, housing and education – compounding social risk factors for health [87].

It is alarming that it has been suggested that people in remote areas with complex needs may need to relocate:

**“**the diversity and level of care and support available in major cities cannot be replicated in very remote areas. In some cases, Indigenous Australians with complex needs will have to move to regional centres or major cities to receive appropriate care and support (as is also the case with non-Indigenous Australians)” [34].

When considered in light of the UK findings [33] that individuals are more likely to experience the promised gains of personalisation when strong support systems are in place, relocating individuals away from such support systems such as kinship, familiarity and community will have serious implications for care outcomes and equity.

While this paper has focused on the example of rural and remote communities, these concerns are also applicable to individuals with rare or low prevalence disabilities that require specific services, resulting in an inability to access appropriate care even within a metropolitan area. It remains unclear how thin markets (and associated lack of choice and control) will be managed.

## Conclusions

The NDIS has the potential to secure gains in the wellbeing and health of hundreds of thousands of Australians, however this can only be achieved with careful attention to the inequities above and others as they arise. Scheme architects have argued that it has the potential to replace a piecemeal and fragmented set of state-determined services with an empowering model of user choice and control. In line with the findings from the Marmot Review, we would expect this to translate into substantial health gains not just through better services but also psycho-social benefits that accrue through increased social inclusion and economic participation [3].

However, without careful attention to both existing inequities and, diversity and difference across populations (e.g. different disability types and different localities) market based have the serious potential to entrench or even widen inequities. As the NDIS is currently set out, this could occur through inefficient or ineffective (i.e. thin) disability markets, market failure in some areas or by imposing conditions on recipients which ultimately undermine their health (such as forced relocation to achieve choice and control). While continued block funding has been offered as one way to prevent this, we argue that this will lead to inequities in choice and control (and therefore health outcomes). Thus, in market based schemes supplementary funding must be offered in the form of service provider support or seed funding (i.e. to encourage providers to move into areas they haven’t previously worked in).

The NDIS is one of the most ambitious personalised funding schemes in the world. As it moves through implementation, the potential for it to adversely impact existing inequities should be monitored and adjustments made such as, for example, continued block funding in remote areas or continued ‘seed’ funding for establishing better services in thin markets. The implementation of NDIS, and it’s impact on health outcomes, holds lessons for any country that looks to implement individualised funding schemes for care provision. Social policies can inadvertently extend health inequities unless consideration is given to these during design and implementation. Policies like the NDIS show that the way that funding is allocated, and not simply the amount of funding allocated, matters for health outcomes.

## Appendix

Australian Department Housing, Community Services and Indigenous Affairs of 2012, *Report to the Council of Australian Governments 2012: laying the groundwork 2011–2014.* , Department of Families, Housing, Community Services and Indigenous Affairs, [Canberra, A.C.T.].

Australian Productivity Commission 2011, *Disability care and support: productivity commission inquiry report* , Productivity Commission, Melbourne, Vic.

Commonwealth Department of Family and Community Services 2014, *Progress Report to the Council of Australian Governments* , Commonwealth Government of Australia, Canberra.

Commonwealth Department of Family and Community Services 2015a, *Proposal for a National Disability Insurance Scheme Quality and Safeguarding framework* , Commonwealth Government of Australia, Canberra.

Commonwealth Department of Family and Community Services 2015b, *Proposal for a National Disability Insurance Scheme Quality and Safeguarding framework: Consultation Paper* , Commonwealth Government of Australia, Canberra.

Department of Social Services 2015, *Integrated Market, Sector and Workforce Strategy* , Commonwealth Government of Australia, Canberra.

Department of Social Services 2016, *National Disability Insurance Scheme Quality and Safeguards Framework: Consultation Report* , Commonwealth of Australia: Canberra.

Department of Social Services 2016, *NDIS Quality and Safeguarding Framework.* Commonwealth of Australia: Canberra.

NDIA 2013a, *Quarterly Report to COAG Disability Reform Council December 2013* , National Disability Insurance Agency, Victoria.

NDIA 2013b, *Quarterly Report to Standing Council September 2013* , National Disability Insurance Agency, Victoria.

NDIA 2014a, *Quality Assurance and Safeguards Working Arrangements* , National Disability Insurance Agency, Victoria.

NDIA 2014b, *Quarterly Report to COAG Disability Reform Council December 2014* , Commonwealth Government of Australia, Victoria.

NDIA 2014c, *Quarterly Report to COAG Disability Reform Council June 2014* , National Disability Insurance Agency, Victoria.

NDIA 2014d, *Quarterly Report to COAG Disability Reform Council March 2014* , National Disability Insurance Agency, Victoria.

NDIA 2014e, *Quarterly Report to COAG Disability Reform Council September 2014* , National Disability Insurance Agency, Victoria.

NDIA 2015a, *A Framework for Information, Linkages and Capacity Building* , National Disability Insurance Agency, Victoria.

NDIA 2015b, *Information, Linkages and Capacity Building Commissioning Framework – Consultation Draft* , National Disability Insurance Agency, Victoria.

NDIA 2015c, *Quarterly Report to COAG Disability Reform Council December 2015* , National Disability Insurance Agency, Victoria.

NDIA 2015d, *Quarterly Report to COAG Disability Reform Council June 2015* , National Disability Insurance Agency, Victoria.

NDIA 2015e, *Quarterly Report to COAG Disability Reform Council September 2015* , National Disability Insurance Agency, Victoria.

NDIA 2016a, *ILC Consultations - Summary Report* , National Disability Insurance Agency, Victoria.

NDIA 2016b, *Integrated Market, Sector and Workforce Strategy* , National Disability Insurance Agency, Victoria.

NDIA 2016c, *Quarterly Report to COAG Disability Reform Council March 2016* , National Disability Insurance Agency, Victoria.

NDIA 2016d, *Quarterly Report to COAG Disability Reform Council September 2016* , National Disability Insurance Agency, Victoria.

*Quarterly Report to COAG Disability Reform Council June 2016* 2016, National Disability Insurance Agency, Victoria.

Whalan, J, Acton, P & Harmer, D 2014, *A review of the capabilities of the National Disability Insurance Agency* , NDIS, Canberra.

## Footnotes

1 There are discrepancies in the documents made available to the public. For example, not all quarterly reports from the National Disability Insurance Agency to the Council of Australian Governments can be found on government websites. Requests to obtain documents were unsuccessful.

## References

[1] Link BG, Phelan J (1995). Social conditions as fundamental causes of disease. J Health Soc Behav.

[2] CSDH. Closing the gap in a generation. Geneva: WHO; 2008.

[3] Marmot M (2010). Fair society, healthy lives: the marmot review. Strategic review of health Inequalitites in England post-2010.

[4] Bambra C, Gibson M, Sowden A, Wright K, Whitehead M, Petticrew M (2010). Tackling the wider social determinants of health and health inequalities: evidence from systematic reviews. J Epidemiol Community Health.

[5] Coburn D (2004). Beyond the income inequality hypothesis: class, neo-liberalism, and health inequalities. Soc Sci Med.

[6] Mackenbach JP (2014). Political determinants of health. The European Journal of Public Health.

[7] Wilikson R, Pickett K (2009). The Spirit level.

[8] Carey G, Crammond B (2015). Action on the social determinants of health: views from inside the policy process. Soc Sci Med.

[9] Baum F (2007). Cracking the nut of health equity: top down and bottom up pressure for action on the social determinants of health. Promot Educ.

[10] Baum F (2011). From norm to Eric: avoiding lifestyle drift in Australian health policy. Aust N Z J Public Health.

[11] Friel S (2014). Inequities in the freedom to lead a flourishing and healthy life: issues for healthy public policy. International Journal of Health Policy Management.

[12] Friel S, Marmot M (2011). Social determinants of global health equity. Annu Rev Public Health.

[13] Baum FE, Laris P, Fisher M, Newman LA, MacDougall C (2014). Dear health minister: tend the garden but make sure you fence the crocodiles. J Epidemiol Community Health.

[14] Baum FE, Laris P, Fisher M, Newman L, MacDougall C (2013). “Never mind the logic, give me the numbers”: former Australian health ministers’ perspectives on the social determinants of health. Soc Sci Med.

[15] Raphael D. Beyond policy analysis: the raw politics behind opposition to healthy public policy. Health Promot Int. 2014; 10.1093/heapro/dau044 24870808

[16] Smith K. Beyond evidence-based policy in public health: Palgrave Macmillan; 2014.

[17] Carey G, Friel S (2015). Understanding the role of public Administration in Implementing Action on the social determinants of health and health inequities. International Journal of Health Policy and Management.

[18] Carey G (2016). Re-Conceptualising public health interventions in government: a response to recent commentaries. International Journal of Health Policy Management..

[19] Carey G, Crammond B. A glossary of policy frameworks: the many forms of “universalism” and policy “targeting.”. J Epidemiol Community Health. 2017:303–7. 10.1136/jech-2014-204311 25294894

[20] Benach J, Malmusi D, Yasui Y, Martínez JM (2013). A new typology of policies to tackle health inequalities and scenarios of impact based on Rose’s population approach. J Epidemiol Community Health.

[21] Frohlich K, Potvin L (2008). The inequality paradox: the population approach and vulnerable populations. Am J Public Health.

[22] World Health Organization (2011). World report on disability.

[23] Bureau of Statistics (2015). Disability, ageing and Carers: summary of findings, 2015.

[24] Kavanagh AM, Krnjacki L, Aitken Z, LaMontagne AD, Beer A, Baker E (2015). Intersections between disability, type of impairment, gender and socio-economic disadvantage in a nationally representative sample of working-aged Australians. Disability and Health Journal.

[25] Emerson E, Llewellyn G, Honey A, Kariuki M (2012). Lower well-being of young Australian adults with self-reported disability reflects their poorer living conditions rather than health issues. Aust N Z J Public Health.

[26] Emerson E, Madden R, Graham H, Llewellyn G, Hatton C, Robertson J (2011). The health of disabled people and the social determinants of health. Public Health.

[27] Mithen J, Aitken Z, Ziersch A, Kavanagh AM (2015). Inequalities in social capital and health between people with and without disabilities. Soc Sci Med.

[28] State Government of Victoria. Victorian population health survey of people with an intellectual disability 2009. Victoria, Australia; 2011.

[29] Esping-Anderson G (1990). Three worlds of welfare capitalism.

[30] Navarro V, Shi L (2001). The political context of social inequalities and health. Soc Sci Med.

[31] Askheim OP (1999). Personal assistance for disabled people–the Norwegian experience. Int J Soc Welf.

[32] Askheim OP, Bengtsson H, Richter BB (2014). Personal assistance in a Scandinavian context: similarities, differences and developmental traits. Scand J of Disability Res.

[33] Needham C (2013). The boundaries of budgets: why should individuals make spending choices about their health and social care? [internet].

[34] Australian Productivity Commission. Disability care and support: productivity commission inquiry report. Melbourne, Vic.: Productivity Commission; 2011.

[35] Hill M, Hupe P. Implementing public policy. Second Edition. London: Sage; 2009.

[36] LeGrand J (2007). Delivering public services through choice and competition: the other invisible hand.

[37] Hood C, Bogdanor V (2005). The idea of joined-up government: a historical perspective. Joined-up government.

[38] Klijn E-H, Koppenjan J (2000). Public management and policy networks. Public Management.

[39] Needham C, Glasby J (2015). Personalisation – love it or hate it?. J of Integr Care.

[40] Williams I, Dickinson H (2016). Going it alone or playing to the crowd? A critique of individual budgets and the personalisation of health Care in the English National Health Service: individual budgets and the personalisation of health care. Aust J of Publ Administration.

[41] Anttonen A, Anttonen A, Haikio L, Stefansson K (2012). Universalism and the challenge of diversity. Welfare state, universalism and diversity.

[42] Riddell S, Pearson C, Jolly D, Barnes C, Priestley M, Mercer G (2005). The development of direct payments in the UK: implications for social justice. Social Policy and Society.

[43] Giaimo S, Manow P (1999). Adapting the welfare state the case of health care reform in Britain, Germany, and the United States. Comp Political Stud.

[44] Gadsby EW. Personal budgets and health: a review of the evidence. London (UK): PRUComm [Internet]. 2013 [cited 2015 Nov 16]; Available from: http://blogs.lshtm.ac.uk/prucomm/files/2013/04/Personal-Budgets-review-of-evidence_FINAL-REPORT.pdf

[45] Gash T, et al. Making public service markets work Professionalising government’s approach to commissioning and market stewardship. 2014 [cited 2016 Jul 4]; Available from: http://www.lgcplus.com/Journals/2013/07/18/q/u/d/Making-public-service-markets-work.pdf.

[46] Needham C, Dickinson H. “Any one of us could be among that number”: Comparing the Policy Narratives for Individualized Disability Funding in Australia and England. Social Policy & Administration [Internet]. 2017 [cited 2017 Jun 5];Online first. Available from: http://doi.wiley.com/10.1111/spol.12320.

[47] Thill C (2015). Listening for policy change: how the voices of disabled people shaped Australia’s National Disability Insurance Scheme. Disability & Soc.

[48] Collings S, Dew A, Dowse L (2016). Support planning with people with intellectual disability and complex support needs in the Australian National Disability Insurance Scheme. J Intellect Dev Disabil.

[49] Carey G, Kay A, Nevile A. Institutional legacies and “sticky layers”: what happens in cases of transformative policy change? Administration & Society 2017;95399717704682.

[50] Walsh J, Johnson S (2013). Development and principles of the national disability insurance scheme. Australian Economic Review.

[51] National Disability Insurance Agency (2017). NDIS performance (NDIS website) [internet].

[52] Whalan J, Acton P, Harmer D (2014). A review of the capabilities of the national disability insurance agency.

[53] Scullion N. NDIS supporting 100,000 Australians. Senator Nigel Scullion (Minister for Disability) Media Release [Internet]. Canberra; 2017; Available from: https://www.nigelscullion.com/media+hub/NDIS+supporting+100%2C000+Australians.

[54] Carey G, Crammond B, De Leeuw E. Towards health equity: a framework for the application of proportionate universalism. Int J Health Equity. 2015;14 10.1186/s12939-015-0207-6 PMC4570091 26369339

[55] Thompson S, Hoggett P (1996). Universalism, selectivism and particularism: towards a postmodern social policy. Critical Social Policy..

[56] Bonyhady B (2014). The NDIS vision: delivering the plan.

[57] NDIS. Overview of the NDIS: Supports [Internet]. 2016 [cited 2016 Mar 10]. Available from: https://www.ndis.gov.au/operational-guideline/overview.html#5.

[58] Soldatic K, van Toorn G, Dowse L, Muir K (2014). Intellectual disability and complex intersections: marginalisation under the National Disability Insurance Scheme. Research and Practice in Intellectual and Developmental Disabilities.

[59] Brennan C, Rice J, Traustadóttir R, Anderberg P (2017). How can states ensure access to personal assistance when service delivery is decentralized? A multi-level analysis of Iceland, Norway and Sweden. Scandinavian Journal of Disability Research.

[60] NDIA. Quarterly report to COAG disability reform council June 2015. Victoria: National Disability Insurance Agency; 2015.

[61] Commonwealth Department of Social Services (2015). Proposal for a National Disability Insurance Scheme Quality and safeguarding framework.

[62] Department of Social Services. In: National Disability Insurance Scheme Quality and Safeguards Framework. Commonwealth of Australia: Consultation Report; 2016.

[63] Department of Social Services (2016). NDIS quality and safeguarding framework.

[64] Department of Social Services (2015). Integrated market, sector and workforce strategy.

[65] LeGrand J, Bartlett W (1993). Quasi-markets and social policy.

[66] Considine M, Lewis JM (2012). Networks and interactivity: ten years of street-level governance in the United Kingdom, the Netherlands and Australia. Public Management Review.

[67] Spicker P (1994). Understanding particularism. Crit Social Policy.

[68] Considine M, Lewis JM, O’Sullivan S (2011). Quasi-markets and service delivery flexibility following a decade of employment assistance reform in Australia. J Social Policy.

[69] Needham C (2010). Debate: personalized public services—a new state/citizen contract?. Public Money & Manage.

[70] Glasby J, Littlechild R (2009). Direct payments and personal budgets: putting personalisation into practice.

[71] Bornat J, Leece J (2006). Developments in direct payments.

[72] McLean G, Sutton M, Guthrie B (2006). Deprivation and quality of primary care services: evidence for persistence of the inverse care law from the UK quality and outcomes framework. J Epidemiol Community Health.

[73] Hart JT (1971). The inverse care law. Lancet.

[74] Piccenna L, Chee M, Lewis V, Gruen RL, Bragge P (2015). Briefing document: Optimising self-directed funding for the long-term disabled.

[75] NDIS (2016). Report on the sustainability of the scheme.

[76] Hart J. The inverse care law. A Sociology of Medical Practice. 1975;189

[77] Lorenc T, Petticrew M, Welch V, Tugwell. What types of interventions generate inequalities? Journal of Epidemiology and Community7. 10.1136/jech-2012-201257 22875078

[78] NDIS (2015). NDIS Price Guide 2015 [Internet]. NDIS Website.

[79] Douglas J, Bigby C, Knox L, Browning M (2015). Factors that underpin the delivery of effective decision-making support for people with cognitive disability. Res Pract Intellectual Dev Disabilities..

[80] O’Connor DB, Jones F, Conner M, McMillan B, Ferguson E (2008). Effects of daily hassles and eating style on eating behavior. Health Psychol.

[81] Sen A (1999). Commodities and capabilities.

[82] Commonwealth Department of Social Services (2015). Proposal for a National Disability Insurance Scheme Quality and safeguarding framework: consultation paper.

[83] Girth A (2012). Outsourcing public service delivery: management responses in noncompetitive markets. Public Administration Research.

[84] NDIS (2016). Victorian market position statement.

[85] NDIA (2016). ILC consultations - summary report.

[86] My Disability Matters. ILC: A Serious Imbalance in the NDIS & Associated Problems [Internet]. My Disability Matters; 2016. Available from: https://mydisabilitymatters.com.au/ndis/ilc-a-serious-imbalance-in-the-ndis/21540/.

[87] Alliance NRH (2014). Income inequality experienced by the people of rural and remote Australia.

